# Microwave Angiography by Ultra-Wideband Sounding: A Preliminary Investigation

**DOI:** 10.3390/diagnostics13182950

**Published:** 2023-09-14

**Authors:** Somayyeh Chamaani, Jürgen Sachs, Alexandra Prokhorova, Carsten Smeenk, Tim Erich Wegner, Marko Helbig

**Affiliations:** 1 Time Domain Electromagnetics Laboratory, Faculty of Electrical Engineering, K. N. Toosi University of Technology, Tehran 16317, Iran; 2 Electronic Measurements and Signal Processing Group, Technische Universität Ilmenau, 98693 Ilmenau, Germany; juergen.sachs@tu-ilmenau.de (J.S.); carsten.smeenk@tu-ilmenau.de (C.S.); tim-erich.wegner@tu-ilmenau.de (T.E.W.); 3 ILMSENS GmbH, 98693 Ilmenau, Germany; 4 Biosignal Processing Group, Technische Universität Ilmenau, 98693 Ilmenau, Germany; alexandra.prokhorova@tu-ilmenau.de

**Keywords:** microwave imaging, angiography, arterial imaging, MIMO ultra-wideband array

## Abstract

Angiography is a very informative method for physicians such as cardiologists, neurologists and neuroscientists. The current modalities experience some shortages, e.g., ultrasound is very operator dependent. The computerized tomography (CT) and magnetic resonance (MR) angiography are very expensive and near infrared spectroscopy cannot capture the deep arteries. Microwave technology has the potential to address some of these issues while compromising between operator dependency, cost, speed, penetration depth and resolution. This paper studies the feasibility of microwave signals for monitoring of arteries. To this aim, a homogenous phantom mimicking body tissue is built. Four elastic tubes simulate arteries and a mechanical system creates pulsations in these arteries. A multiple input multiple output (MIMO) array of ultra-wideband (UWB) transmitters and receivers illuminates the phantom and captures the reflected signals over the desired observation time period. Since we are only interested in the imaging of dynamic parts, i.e., arteries, the static clutters can be suppressed easily by background subtraction method. To obtain a fast image of arteries, which are pulsating with the heartbeat rate, we calculate the Fourier transform of each channel of the MIMO system over the observation time and apply delay and sum (DAS) beamforming method on the heartbeat rate aligned spectral component. The results show that the lateral and longitudinal images and motion mode (M-mode) time series of different points of phantom have the potential to be used for diagnosis.

## 1. Introduction

Current non-invasive modalities for vascular imaging include computerized tomography angiography (CTA), magnetic resonance angiography (MRA), positron emission tomography (PET), and ultrasonography [1]. CTA and PET use ionizing X-ray radiation; therefore, it is harmful to the human body and it is not recommended to be utilized multiple times. MRA and CTA do not provide information about hemodynamics (blood flow) [2]. Hemodynamic information may eventually assist in risk stratification schemes of different diseases [3]. Doppler ultrasound can be employed for blood flow measurement, but it is limited due to small field of view, operator expertise, and sensitivity to a poor acoustic window. Accordingly, building a portable device that provides vascular images from the body is very promising. Unlike traditional computerized tomography (CT) and magnetic resonance imaging (MRI) monitoring units, such devices can be employed in ambulances and helicopters for emergency cases. Portable imaging devices can also be employed in the intensive care unit for continuous monitoring of critical patients. Currently, patients are moved from the Intensive Care Unit to CT, PET or MRI unit for discrete monitoring when necessary. Conventional monitoring not only provides limited information on disease progression, but also creates additional risk.

One alternative modality that can provide the required temporal resolution for hemodynamic measurement is microwave-based sensing and imaging. This technology utilizes very low power transceivers and is non-ionizing, low cost and portable.

The advantages of using microwave frequencies for body imaging have been recognized by the research community over the past decade and different groups around the world reported studies on static 3D microwave imaging.

In a paper by Person et al. about stroke detection using microwaves [4], an ultra-wideband (UWB)-based array mounted on a helmet is used for stroke diagnosis for pre-hospital applications. Recently, an operational version of this helmet has been released for emergency ambulances and helicopters [5]. This helmet consists of 16 antennas and therefore has a limited resolution. The proposed solution to improve the detection performance was using machine learning algorithms that are trained by MRI or CT-scans of healthy people and stroke patients [5]. However, this method only detects and classifies the type of stroke and provides neither the 3D nor the vascular image of the brain.

Another research group in Australia reported a preclinical prototype device for microwave brain imaging [6]. This group is also focused on static 3D imaging.

A device with 200 antennas on the base of multiport vector network analyzer method is proposed by Poltschak et al. [7]. A limited number of clinical trials with this structure have been recently released. This system uses a vector network analyzer, which has a higher price, and longer recording time compared to UWB time-domain transceivers. This system also focuses on static 3D and neurofunctional imaging, i.e., it does not provide vascular images and hemodynamic.

In a study by Razzicchia et al. about using metamaterial for microwave brain imaging [8], it is proposed to enhance the strength of the received signal from intracranial bleeding for microwave imaging using a metamaterial. The implemented metamaterial enhanced the detection and localization accuracy of bleeding in a static 3D image.

Lauteslager et al. have been working towards impulse UWB radar techniques for neuro-functional imaging. They monitor intracranial heartbeat and neurological activities using UWB-based sensors [9]. Their sensors are also used for mental stress classification [10]. They use multiple input multiple output (MIMO) arrays of UWB sensors for imaging from dynamic parts of the body such as heart [11] and the artery inside the thigh [12,13].

In this paper, a preliminary investigation about vascular imaging using a MIMO array of UWB sounders is conducted. The type of UWB signal is a maximum-length binary sequence (M-sequence). To prove the concept, a pulsation system is implemented to mimic arterial pulsations. An elastic hose simulates the artery. This hose is placed inside an oil–gelatin phantom [14]. The phantom is illuminated by the MIMO array including 256 channels. The output signals of MIMO radar create a 3D matrix, i.e., range, observation time and channel. We calculate the Fourier transform of each channel regarding the observation time. Therefore, the new 3D matrix dimensions are range, frequency and channel. Provided that the pulsation frequency is known, which is available by electrocardiogram in practical scenarios, this 3D data matrix of MIMO system is reduced to a 2D matrix: range channel at the pulsation frequency. Applying the delay and sum (DAS) algorithm on the obtained 2D matrix, the image of pulsating vessels (arterial image) is calculated very fast. In the experimental setup, four arteries in the phantom are considered. Six scenarios are tested: in the first five scenarios, the tubes are filled with air, and in the last scenario, the tubes are filled with water. The results show that at least three arteritis are always detectable. The deepest artery, which is 4.5 cm below the surface, is not detectable in the sixth scenario. Moreover, the motion-mode (M-mode) time series of different voxels are calculated and are consistent with the scenarios. The M-mode comes from medical ultrasound terminology and documents tissue movements [15].

To the best of our knowledge, the only works considered vascular microwave imaging are those of Lauteslager et al. [9,12,13], without any validation/comparison of the obtained image with the real placement of artery. Using a pulsating phantom, our work proves the feasibility of UWB signals for artery pulsation monitoring. Moreover, our applied dynamic imaging algorithm is much faster than the algorithm implemented in [9,12,13].

## 2. Materials and Methods

### 2.1. Scattering Model

#### 2.1.1. Scattering from Static Target

**Figure 1:**
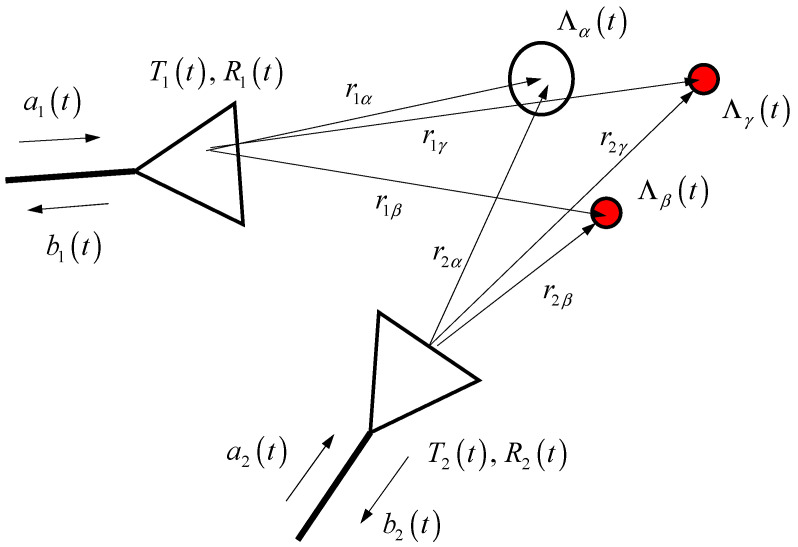
A typical microwave imaging setup. Usually in an MIMO system, the number of antennas is more than two.

We assume far field conditions even if this may not always be valid and the artery is modelled as a simple cylinder. To keep the type of scattering effect, we consider a simple case: a long bone (cylinder α) and a long artery (cylinder β, γ) (Figure 1).

Neglecting angular dependencies, polarization, propagation loss, etc., and according to the time-domain Friis formula and radar equation, the signal scattered from, e.g., the bone (index *κ*) measured at port *j* when considering the *i*th antenna as the transmitter, is

(1) $$\begin{array}{ll} \underset{received\;signal}{\underbrace{b_{ji,\kappa }\left(t\right)}} & =\underset{stimulus}{\underbrace{a_{i}\left(t\right)}}*\underset{radar\;path\;impulse\;response}{\underbrace{T_{i}\left(t\right)*\frac{\delta \left(t-\frac{r_{i\kappa }}{c}\right)}{2\pi r_{i\kappa }}*\Lambda_{\kappa }\left(t\right)*\frac{\delta \left(t-\frac{r_{j\kappa }}{c}\right)}{2\pi r_{j\kappa }}*R_{j}\left(t\right)}}\\ & =a_{i}\left(t\right)*g_{ji,\kappa }\left(t\right)*\Lambda_{\kappa }\left(t\right) \end{array},$$

where δ(.) shows the Dirac delta function, *t* shows propagation time which is of the order of nanosecond, $\Lambda_{\kappa }(.)$ shows the scattering impulse response function (IRF) of scatterer κ (see Appendix A), and distances $r_{\ldots }$ refer to the geometric center of the antennas or targets. $T_{i}(t),\;R_{j}(t)$ show impulse response function (IRF) of the *i*th antenna in the transmission mode and IRF of the *j*th antenna in the reception mode, respectively, and ∗ shows the convolution operator. To shorten the notation, the antenna and propagation path behavior are joint in $g_{ji,\kappa }\left(t\right)$. In the case of multiple scatterers (k scatterers) and if we assume that all transmitters are stimulated with identical signals, the total received signal is

(2) $$b_{ji}\left(t\right)=a\left(t\right)*\sum_{\kappa }^{}g_{ji,\kappa }\left(t\right)*\Lambda_{\kappa }\left(t\right).$$

If the sounding signal is a short pulse $a\left(t\right)=\delta \left(t\right)$, the received signal is directly proportional to the radar path impulse response since convolution with the Dirac function does not change the result. In the case of an arbitrary wideband signal as, e.g., an M-sequence, $a\left(t\right)=m\left(t\right)$, one has to perform an additional pulse compression:

(3) $$y_{ji}\left(t\right)=b_{ji}\left(t\right)*m\left(-t\right)=\underset{\approx \delta \left(t\right)}{\underbrace{m\left(t\right)*m\left(-t\right)}}*\sum_{\kappa }^{}g_{ji,\kappa }\left(t\right)*\Lambda_{\kappa }\left(t\right)\approx \sum_{\kappa }^{}g_{ji,\kappa }\left(t\right)*\Lambda_{\kappa }\left(t\right),$$

which finally also leads to a signal which is proportional to the impulse response of the radar path. The advantage of the latter approach is a better noise and jitter performance (see [16]).

Similar formulas can be written for scattering from other tubes. However, for a pulsating artery, the reflected signal is a function of blood pressure and is therefore time variant as is explained in the following part.

#### 2.1.2. Scattering from Pulsating Artery

**Figure 2:**
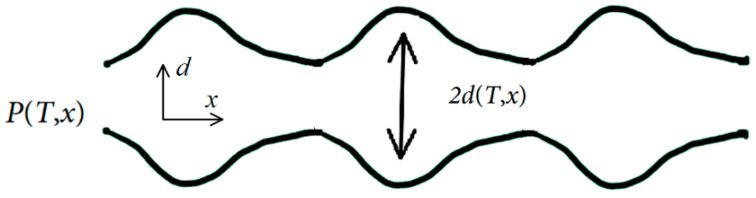
Oscillatory flow in an elastic tube.

Since the arteries have elasticity [17], the pressure pulse produced by the heart cycle results in inflation and deflation along the artery (see Figure 2). These inflations, which are present in elastic tubes—not in rigid tubes—reduce the speed of flow inside the artery. However, they store some part of energy in the inflated part of the tube. This energy is similar to the energy stored within a compressed or extended spring and is recoverable. The stored energy is recovered as the walls of the tube “recoil” during the diastolic phase of the oscillatory cycle. As pressure rises at one end of the tube, the tube inflates locally and then deflates as the pressure subsides. As it deflates, it pushes fluid forward along the tube. In fact, it pushes the local inflation of the tube forward, thus forming a wave motion along the tube [17].

As explained above, inflation and deflation change the radius of the artery depending on the stiffness/elasticity of the artery.

If we consider a cylinder, which varies a bit, and its radius $d=d_{0}+\Delta d$, we can approximatively write

(4) $$\Lambda \left(t\right)\approx \Lambda_{0}\left(t\right)+\Delta d\cdot \Pi \left(t\right).$$

$\Pi \left(t\right)$ is a function describing the waveform modifications of the incident wave vs propagation time *t* (see Appendix A). The radius of the artery varies depending on its stiffness (K) and pressure *P*:

(5) $$\Delta d=\frac{\Delta P}{K}.$$

If a time variable pressure (e.g., sinusoidal with frequency ν) over observation time *T* subjects an artery,

(6) $$P=P_{0}+\Delta P\cos\left(2\pi \nu T\right).$$

The reflection coefficient can be written as (see Appendix A)

(7) $$\Lambda \left(t,T\right)\approx \Lambda_{0}\left(t\right)+\frac{1}{K}\Delta P\cos\left(2\pi \nu T\right)\cdot \Pi \left(t\right).$$

That is, the reflections become additionally depended on the observation time T. By repeating the radar measurements over a longer observation time T, we finally obtain a two-dimensional data set

(8) $$y_{ji}\left(t,T\right)\approx \sum_{\kappa }^{}g_{ji,\kappa }\left(t\right)*\left(\Lambda_{0\kappa }\left(t\right)+\frac{1}{K_{\kappa }}\Delta P\cos\left(2\pi \nu T\right)\Pi_{\kappa }\left(t\right)\right).$$

Finally, these signals, which are captured over all channels of the antenna array, are used for image reconstruction, which is described in the next section.

### 2.2. Imaging

**Figure 3:**
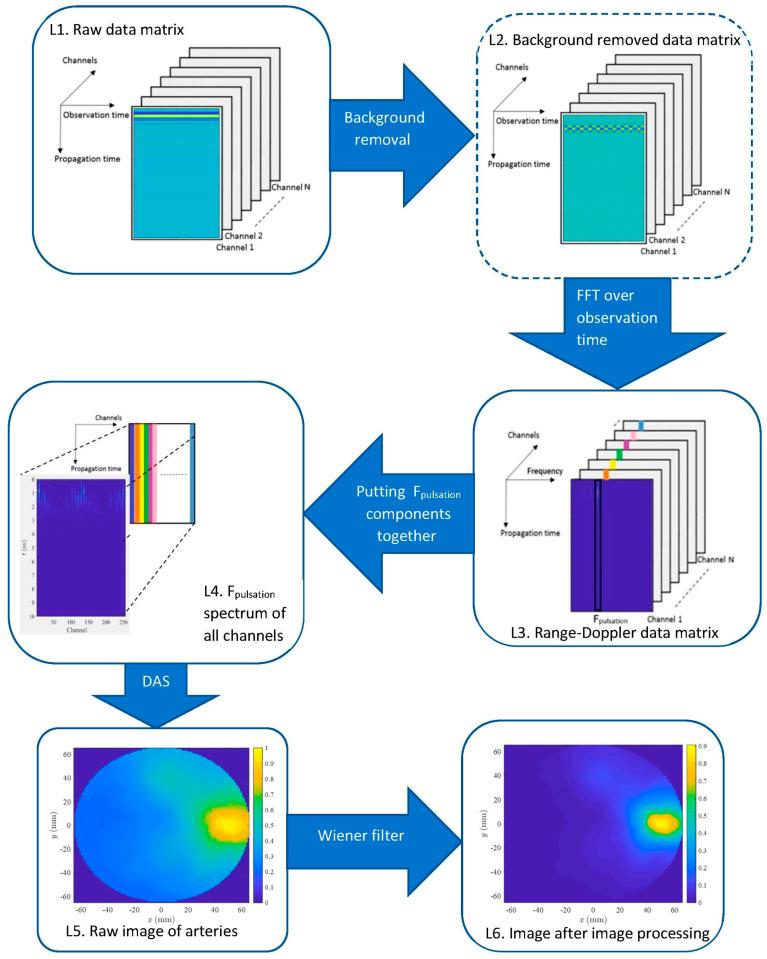
Pipeline of imaging algorithm.

The pipeline of the imaging algorithm is shown in Figure 3. Data matrix in 6 different stages of algorithm is shown by L1–L6, respectively. The following subsections describe the process.

#### 2.2.1. Preprocessing: Background Removal

For vascular imaging, since we are only interested in pulsating parts (arteries), we can easily use the concept of differential imaging [16]. In differential imaging, before image formation, the static-related backscattered signals are removed by background subtraction. There are many methods for background subtraction. We utilize average subtraction as follows:

(9) $${\tilde{y}}_{ij}(t,T)=y_{ij}(t,T)-{\bar{y}}_{ij}\left(t\right),$$

where ${\bar{y}}_{ij}\left(t\right)$ is the average of $y_{ij}(t,T)$ over the observation time.

(10) $${\bar{y}}_{ij}\left(t\right)=\frac{1}{T_{I}}\int_{T=0}^{T_{I}}y_{ij}\left(t,T\right)dT.$$

Preferentially, the average value is determined over an integer number of pulsations. Since the reflected signal from the static objects does not change over the observation time, the average of $y_{ij}(t,T)$ includes their response while the average of pulsations is approximately zero. Therefore, ${\tilde{y}}_{ij}(t,T)$ includes only the artery pulsations and other possible nonstationary clutters; the latter one we ignore for the sake of simplicity in this preliminary study. The background removed data matrix is shown by L2 in Figure 3. If we are only interested in the 2D image of arteries but not the M-mode signal, this stage might be removed from the pipeline (see Section 2.2.2).

#### 2.2.2. 2D Range-Doppler Matrix Reconstruction

There are two methods to calculate the image of arteries: 1: Applying beamforming at each observation time sample; calculating the FFT of images over observation time (spectral images) and finally mapping the spectral image at pulsating frequency, and 2: Applying FFT over the observation time to each channel (constructing the 3D range-doppler matrix; L3 in Figure 3); constructing a new 2D matrix by putting the spectral components of each channel at the pulsating frequency ($F_{P}$) together, and finally applying the beamforming method on the new 2D matrix (L4 in Figure 3). The first method needs to apply beamforming N times (N is the number of observation time samples), whereas the second method only needs a single application of beamforming, which is obviously much faster than the former method; therefore, we implement the latter method (see Section 2.2.3). Since we are only interested in image at the pulsation frequency, and static background lies in the direct current component, background removal is not necessary for image reconstruction.

#### 2.2.3. Delay and Sum of MIMO Signals at the Pulsation Frequency

To reconstruct the image of the region of interest (ROI) from the measured received signals, several beamforming methods exist. In this paper, the delay and sum method is implemented.

A. Conventional Delay and Sum

The ROI is divided into a grid of voxels, assigned by their position vector $r_{v}$. To calculate images at each voxel ($I(r_{v})$), the signals of each channel of the MIMO array are shifted with the delays corresponding to the related voxel–antenna distance ($\tau_{v}$) and summed. Then, these shifted and summed signals are trimmed by a windowing/gating signal $w\left(t\right)$. Attenuation compensation is also applied to correct the amplitude of each voxel due to the spreading loss in the phantom. This is achieved by multiplying function $g_{a}$, and finally image $I(r_{v})$ is obtained as in Equation (11) [18].

(11) $$\begin{array}{l} I(r_{v})=\int_{t}w(t){\left(\sum_{j=1}^{k_{T}}\sum_{i=1}^{k_{R}}g_{a}(r_{v})\cdot {\tilde{Y}}_{ij}(t+\tau_{v},F_{p})\right)}^{2}dt\\ {\tilde{Y}}_{ij}(t,F)=FFT({\tilde{y}}_{ij}(t,T))\\ \tau_{v}=\frac{\left|r_{i}-r_{v}\right|+\left|r_{j}-r_{v}\right|}{c}\\ g_{a}=\sqrt{\left|r_{i}-r_{v}\right|\left|r_{j}-r_{v}\right|} \end{array},$$

where $k_{R},\;k_{T}$ are the number of receivers and transmitters, respectively. To calculate delays $\tau_{v}$, the wave speed (or permittivity of ROI) must be known. $r_{i}$ and $r_{j}$ show the position vector of antenna *i* and *j*, respectively. For $w\left(t\right)$, a rectangular window length of 4 samples is used. This value corresponds to $\frac{\lambda }{2}$ at the center frequency of the MIMO radar (3.25 GHz). However, this value is accurate when the antenna’s impulse response is narrow enough. In general, the window length can be dictated by the antenna impulse response and its value is a trade-off between the contrast and the resolution of the image. The impulse response in our setup is about 10 samples (rectangular window). We examine window lengths between 4 and 10 samples and not a big difference in the appearance of the images is observed. The voxel size is 1 mm^3^.

B. Approximated Delay and Sum

Instead of Equation (11), we apply a first-order approximation of DAS with the following formula:

(12) $$\begin{array}{l} I(r_{v})=\int_{t}w(t){\left(\sum_{j=1}^{k_{T}}\sum_{i=1}^{k_{R}}g_{a}(r_{v})\cdot \left|{\tilde{Y}}_{ij}(t+\tau_{v},F_{p})\right|\right)}^{2}dt\\ {\tilde{Y}}_{ij}(t,F)=FFT({\tilde{y}}_{ij}(t,T))\\ \tau_{v}=\frac{\left|r_{i}-r_{v}\right|+\left|r_{j}-r_{v}\right|}{c}\\ g_{a}=\sqrt{\left|r_{i}-r_{v}\right|\left|r_{j}-r_{v}\right|} \end{array},$$

where the image is obtained by delay and sum of absolute value of pulsation spectral components instead of the complex value of it. Formula (12) is more robust than (11), i.e., although with lower resolution, the pulsating target is visible at a more or less correct position. On the contrary, (11) provides better resolution but also a higher probability that, due to different phases, complex values add up destructively and cancel each other.

#### 2.2.4. Post-Processing: Wiener Filter

To reduce the noise/blur in the obtained image, the 2D adaptive noise filter Wiener2 from MATLAB 2018b was applied. We set the neighborhood size as [5 5]. Finally, to emphasize the largest values, we took the power of 3 from the Wiener2 filtered image.

### 2.3. Experimental Setup

#### 2.3.1. Phantom

**Figure 4:**
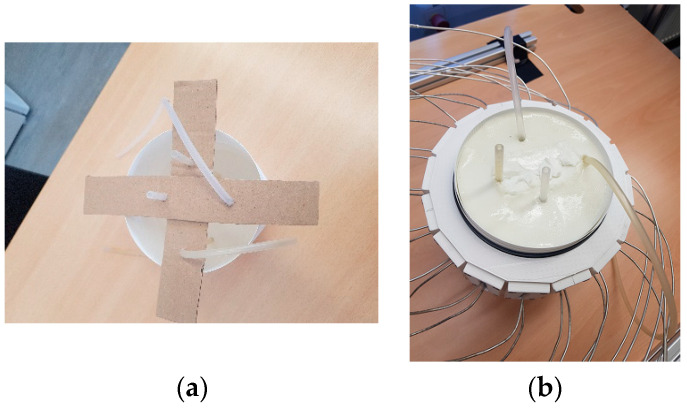
Silicon hoses passing through the bowl: (**a**) before filling with tissue mimicking material; (**b**) after filling the bowl with tissue mimicking material.

**Figure 5:**
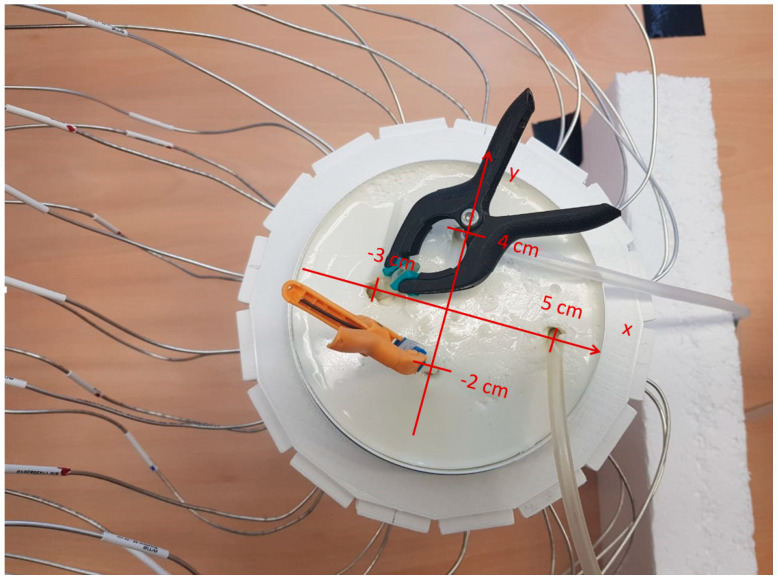
Coordinates of the 4 tubes mimicking the arteries.

**Figure 6:**
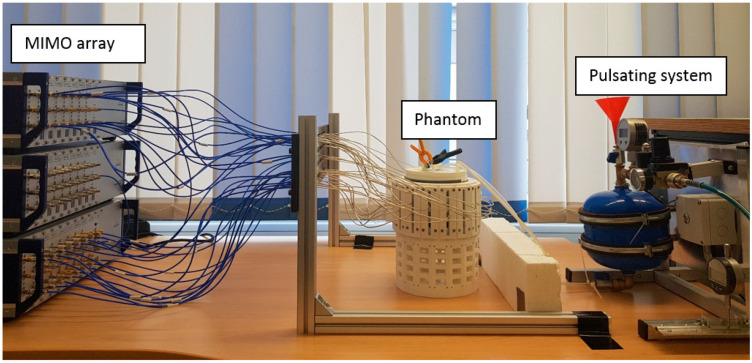
MIMO arterial imaging setup.

Two silicone hoses pass through a plastic bowl, make two U-shaped turns beneath the bowl, and return to the bowl (Figure 4a). Therefore, they mimic four arteries inside the body tissue. The diameter of the hoses is 5 mm. To simulate the body tissue, an oil-gelatin mixture with 40% oil content is used (Figure 4b). This phantom material was developed by Lazebnik et al. [14]. The bowl has a cylindrical shape with a ca. 13 cm diameter, and a 10 cm height. The permittivity of phantom material at the center frequency (3.25 GHz) is ca. 30. In Figure 5, the ends of the hoses placed at (5 cm, 0 cm) and (0 cm, 4 cm) are connected by a T- junction to the pulsating system (Figure 6). The other ends of the hoses placed at (−3 cm, 0 cm) and (0 cm, −2 cm) are closed to create a standing wave in the hoses. If we want to remove any of arteries from the scenario, we put clips in proper positions to cut the flow in the specified artery.

#### 2.3.2. MIMO Radar System

**Figure 7:**
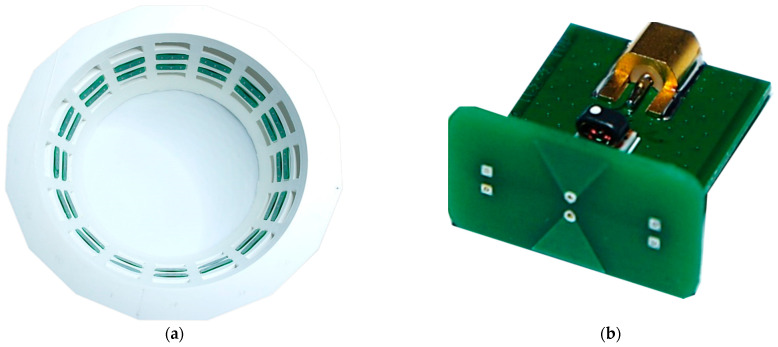
(**a**) Cylindrical mold including the sensing antennas. The phantom is placed inside this mold; (**b**) small passive bowtie antenna.

The UWB MIMO radar device is developed and manufactured by Ilmsens GmbH [19]. Its bandwidth is 6.5 GHz. The antenna array is customized from hyperthermia monitoring system in the laboratory of medical microwave sensing in Ilmenau University of Technology [20]. In this measurement, it includes 16 transmitters and 16 receivers, i.e., 256 channels. The antenna array mold is shown in Figure 7a. The 32 antennas are placed in two rings at heights of −4 cm and −6 cm. The antennas are passive small bow-tie antennas [19,21] shown in Figure 7b.

#### 2.3.3. Pulsation System

**Figure 8:**
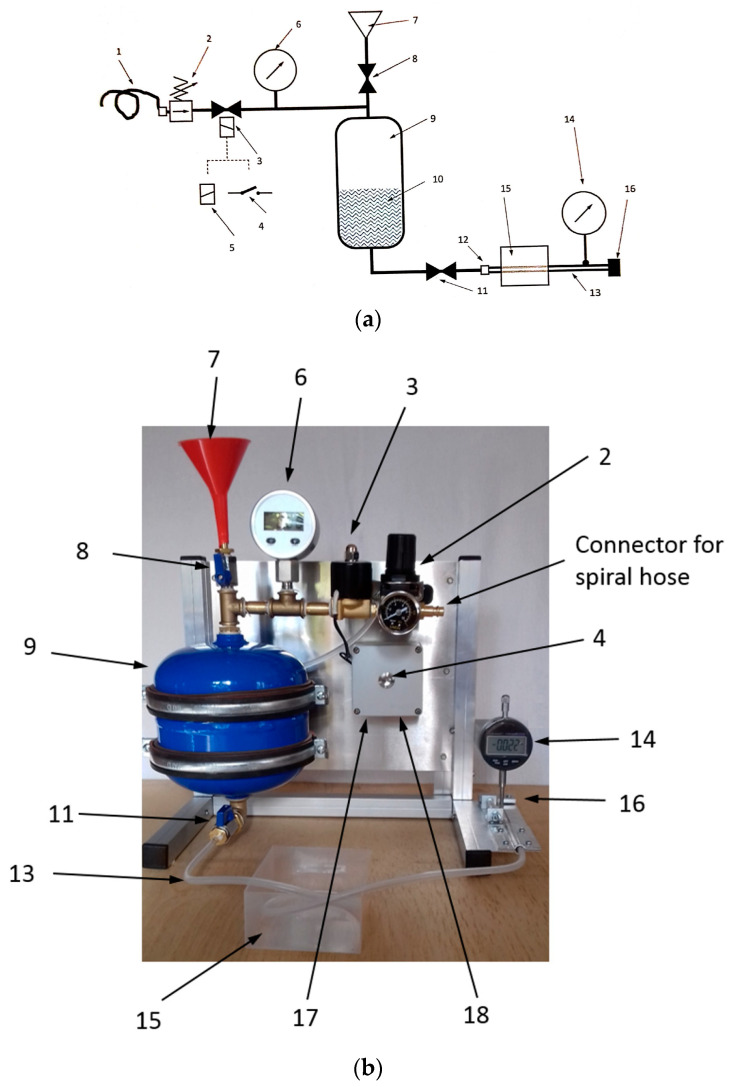
(**a**) Schematic of pulsation system, (**b**) photo of pulsation system. 1: compressed air spiral hose, 2: pressure reducer, 3: magnetic valve, 4: button for magnetic valve, 5: USB relay for magnetic valve, 6: pressure gauge, 7: hopper, 8: hand valve (ball valve), 9: pressure vessel, 10: liquid (blood surrogate), 11: hand valve (ball valve), 12: hose nozzle, 13: silicone hose (artery surrogate), 14: micrometer gauge, 15: container with tissue surrogate, 16: hose clamp, 17: USB-port, and 18: 12V/2A-power supply port.

The system which mimics the arterial pulsation is manufactured by Ilmsens GmbH [19]. The artery’s surrogate is a silicone hose. The variation of the diameter is measured via a micrometer gauge. The measure radial variations during the pulsation are less than 0.2 mm. The schematic and image of the pulsation system are shown in Figure 8. The pulsation system is connected to a compressed air source through a spiral hose (1 in Figure 8). To simulate the heartbeat, the artery pressure has to be modified as a function of time. For that purpose, the magnetic valve (3 in Figure 8) should be activated repetitively over a short time via a relay controlled through USB (5, 17 in Figure 8). The USB is connected to the computer and controlled using a batch file. Additionally, the valve (8 in Figure 8) is partially opened so that the pressure inside the vessel decreases during the period while the magnetic valve (3 in Figure 8) is closed. The time constant of charging the vessel is given by the flow resistance of the air supply and Valve 8 as well as the “storage capacity” of the vessel. The discharging time constant is determined from the flow resistance of Valve 8 and the “storage capacity” of the vessel. The “storage capacity” of the vessel can be modified by the liquid level (10 in Figure 8)—more liquid → low capacity; less liquid → high capacity. The flow resistance of Valve 8 depends on the angular position of its lever handle.

## 3. Results

**Figure 9:**
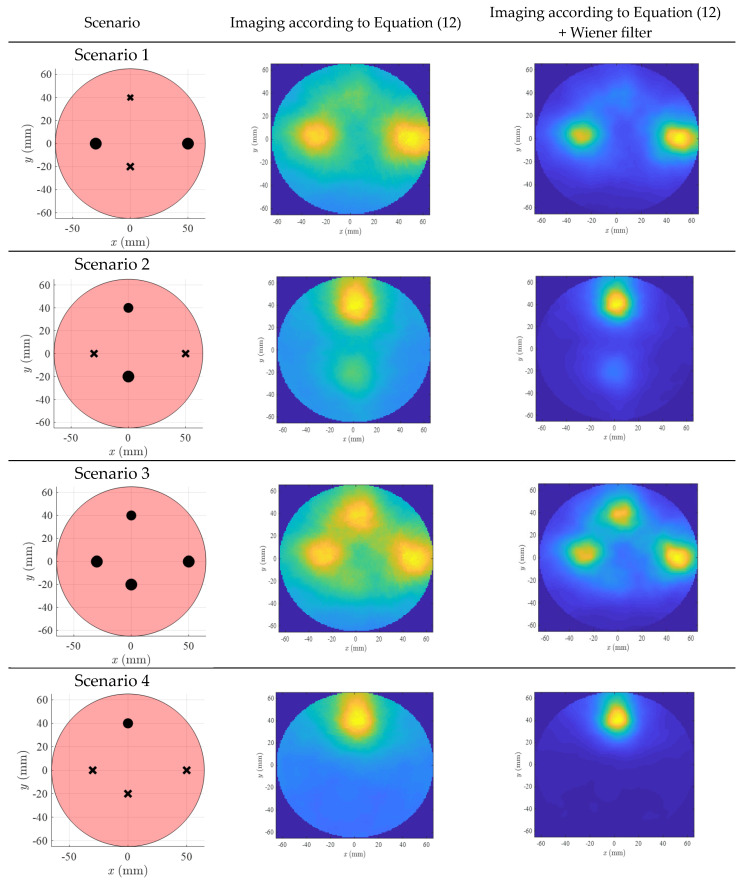

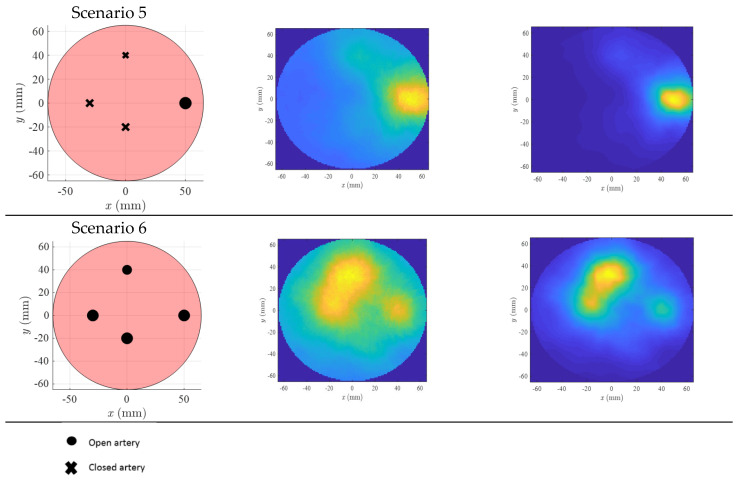
Lateral image of different scenarios.

**Figure 10:**
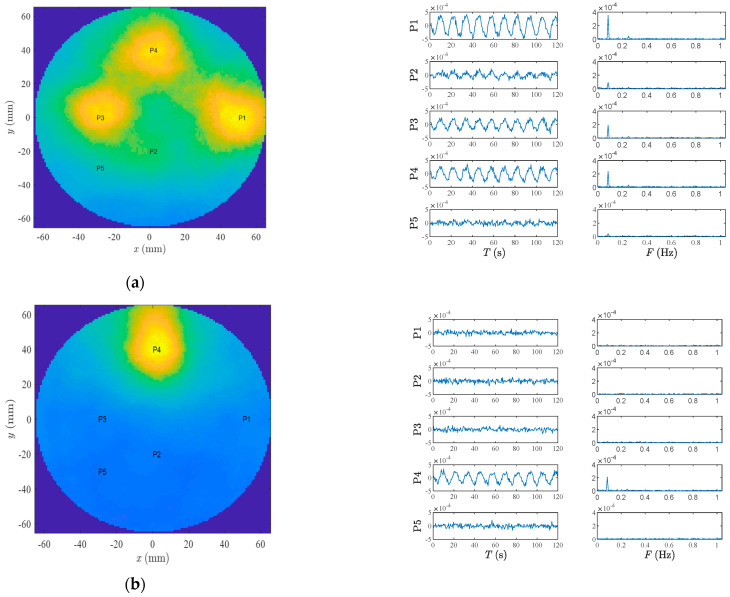

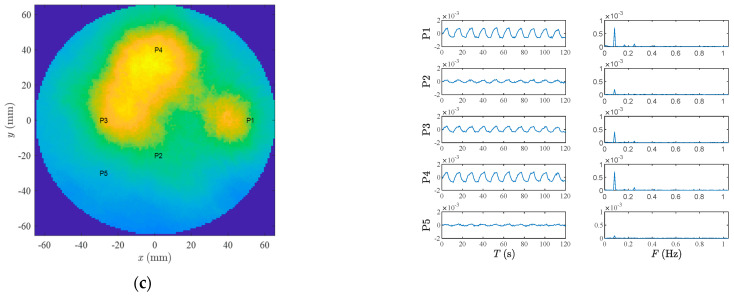
M-mode time series and spectra of some exemplary points for (**a**) Scenario 3, (**b**) Scenario 4, and (**c**) Scenario 6 confirm the plausibility of the corresponding lateral images.

**Figure 11:**
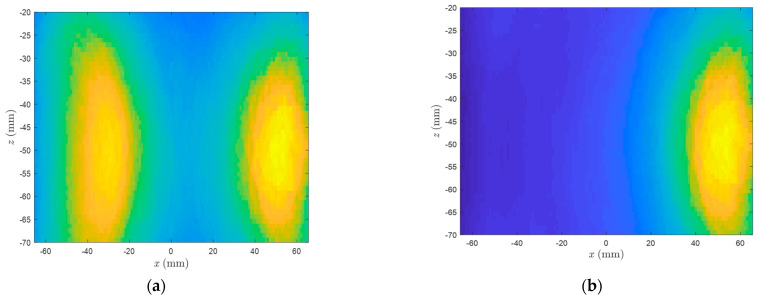
The longitudinal view of arterial images at y = 0 cm: (**a**) Scenario 1 and (**b**) Scenario 5.

The images of six scenarios in a lateral view are plotted in Figure 9. The images are calculated at *z* = −5 cm. In Scenarios 1 to 5, the arteries are filled with air, while in the sixth scenario, the arteries are filled with water. The images correspond to the pulsation frequency (0.08 Hz). Additionally, the M-mode time series in different points are plotted in Figure 10. The M-mode signals are obtained by the delay and sum of received signals at the desired locations. The spectrum of the M-mode signals is also shown in Figure 10. The longitudinal view of arterial images is shown in Figure 11. For the sake of brevity, only Scenarios 3 and 4 are shown. The image plane is y = 0.

## 4. Discussion

As observed in Figure 9, three of the arteries (maximum distance between artery and antenna = 3.5 cm) are easily detectable. The artery placed in (0 cm, −2 cm) is not easily visible in the lateral images. However, when the M-mode signals are investigated (Figure 10a,b), the pulsation on this deep artery is also visible. Scenario 6, which has water-filled arteries, shows a stronger amplitude (can be observed in Figure 10). This scenario is more similar to the real scenarios in which blood circulates in the artery. Compared to Scenario 3, the spatial resolution is worse. However, similar to Scenario 3, still, three arteries are detectable. A precise argument about resolution of this scenario needs more tests, preferably with blood-mimicking materials instead of water.

As shown in Figure 10, the amplitude of M-mode time series in the points free of artery, e.g., P5 in all plots and P1, P2, P3 and P5 in Scenario 4, is very small.

As observed in Figure 11 for longitudinal images, the arteries are visible at a depth between 3 cm and 7 cm, which corresponds to the z position of MIMO antennas. Such a longitudinal image has a potential to investigate arteries to find a possible occlusion which is very common in peripheral artery disease. In other words, this setup with its current resolution has the potential to be used for peripheral artery disease imaging. According to [22], listing measured diameter values of lower-limb arteries of males between ca. 10 mm (proximal femoral artery) and ca. 4 mm (tibial/fibular arteries), our measurement setup (phantom diameter 13 cm, artery imitation diameter 5 mm) anatomically adequately imitates a leg examination. Our results indicate that the pulsation of these peripheral arteries is detectable and localizable. In order to fulfill also the physiological requirements (realistic pulse frequency), it is needed to increase the sampling rate to that several times of the heartbeat, which is feasible. Compared to full images, the required time for calculating the M-mode series is much smaller and, therefore, they can provide information about the artery movement in real time [23].

## 5. Conclusions

The feasibility of microwave technology as a new modality for angiography was studied. A phantom including pulsating hoses which mimicked arteries was implemented. The phantom was illuminated by an MIMO array of UWB sensors. Lateral, longitudinal images of arteries and M-mode time series were reported. At least three of four arteries placed in phantom were easily detectable. The M-mode time series, which provide the hemodynamic information of different points, were consistent with the implemented scenarios. The current setup is satisfactory for large vessels. For example, it can be a promising modality for peripheral artery disease diagnosis where its superiority to ultrasound modality is in lower dependency on operator’s expertise.

Future works should concentrate on the acceleration of data acquisition and processing towards realistic pulse frequencies while maintaining the high number of channels. Furthermore, the optimal bandwidth, the achievable resolution, shorter impulse response of antennas, the minimum detectable artery size, and the penetration depth should be addressed in the future steps. Another area of improving arterial images is the use of machine learning algorithms in order to obtain a more accurate longitudinal image showing the morphology of artery. However, the application of machine learning requires an extensive clinical dataset containing microwave images compared with gold standards.

## Data Availability

Not applicable.

## Appendix A

The scattering is symbolized by the scattering IRF $\Lambda \left(t\right)$ (unit (m/s)). Its Fourier Transform is the scattering length $\underline{\Lambda }\left(f\right)$, which relates to the radar cross-section by (see [24] (Equation 4.64))

$$\sigma =4\pi \underline{\Lambda }{\underline{\Lambda }}^{*}.$$

Scattering at a perfect electric conducting cylinder has been investigated in [25]. We can distinguish three types of scattering:

Rayleigh scattering: the rise time of the sounding pulse is larger than the circumferential propagation time (creeping waves). The radar cross-section per unit length for an infinite cylinder with radius *d* is given by (Equation 2.44 in [25])

$$\sigma =\frac{3}{4}\pi^{2}{\left(\frac{2\pi f}{c}\right)}^{3}d^{4}.$$

Therefore, the radar cross-section is

$$\underline{\Lambda }\left(f\right)=\frac{3\sqrt{\pi }}{4}\frac{d^{2}}{\sqrt{c^{3}}}\sqrt{{\left(2\pi f\right)}^{3}}\;\to \;\Lambda \left(t\right)=\frac{3}{4}d^{2}\sqrt{\frac{\pi }{c^{3}}}u_{3/2}\left(t\right)=\frac{3}{4}\frac{A}{\sqrt{\pi c^{3}}}u_{3/2}\left(t\right),$$

where $u_{2/3}\left(t\right)$ represents a fractional derivation (see [26]).

Hence, the time domain Rayleigh scattering can be expressed by

$$\Lambda \left(t\right)=\frac{3}{4}\frac{A}{\sqrt{\pi c^{3}}}u_{3/2}\left(t\right),$$

in which *A* is the cross-section of the cylinder. That is, the scattering changes the shape of the incident signal always in the same way (fractional derivation of the incident wave), but the magnitude of scattering depends on the cross-sections of the artery.

Optical scatting: the rise time of the sounding pulse is much shorter than the circumferential propagation time. The reflected wave is a mirrored version of the incident one, so that we can approximately write

$$\Lambda \left(t\right)\approx b\;\delta \left(t-\frac{2d}{c}\right),$$

where parameter *b* depends on the dielectric contrast. Note the distances used in Equation (1) in the main text; refer to the center of the objects. Since the scattering takes place at the surface of the artery, the actual traveling path is 2*d* shorter than supposed in Equation (1) in the main text.

Mie or resonant scattering: the rise time is somewhere in between. The time shape and strength of the reflected wave is determined by the target, i.e., it depends on the diameter of the artery. Since arteries are embedded in a lossy medium, resonance effects (creeping waves), however, hardly develop.

If we consider a cylinder, the radius of which varies a bit, $d=d_{0}+\Delta d$, we can approximatively write

$$\Lambda \left(t\right)\approx \Lambda_{0}\left(t\right)+\Delta d\cdot \Pi \left(t\right).$$

Π(t) is a functional describing the waveform modifications of the incident wave. In the case of a thin artery (Rayleigh scattering), it is $\Pi \left(t\right)=\frac{3}{4}\frac{A}{\sqrt{\pi c^{3}}}\;u_{3/2}\left(t\right)$, and in the case of a thick artery (geometric scattering), it describes a simple time shift, which can be approximated by $\Pi \left(t\right)=-\frac{2b}{c}u_{1}\left(t\right)$. The waveform modifications are in both cases simply caused by differentiation (ordinary or fractional). This indicates that the high-frequency content of the radar signal dominates the sensitivity with respect to motion detection.

Elastic artery with quasi-static pressure: If a time variable pressure (e.g., sinusoidal with frequency ν) over time *T* subjects an artery,

$$P=P_{0}+\Delta P\cos\left(2\pi \nu T\right),$$

the radius of the artery varies depending on its stiffness

ΔP=K·Δd.

Hence, for an artery subjected to temporal pressure variation (e.g., sinusoidal with frequency ν), the reflections coefficient may be written as

$$\Lambda \left(t,T\right)\approx \Lambda_{0}\left(t\right)+\frac{1}{K}\Delta P\cos\left(2\pi \nu T\right)\cdot \Pi \left(t\right).$$

That is, the reflections become additionally depended on observation time T. By repeating the radar measurements over a longer time period T, we finally obtain a two-dimensional data set

$$y_{ji}\left(t,T\right)\approx \sum_{\kappa }^{}g_{ji,\kappa }\left(t\right)*\left(\Lambda_{0\kappa }\left(t\right)+\frac{1}{K_{\kappa }}\Delta P\cos\left(2\pi \nu T\right)\Pi_{\kappa }\left(t\right)\right).$$

Herein, $K_{\kappa }$ represents the stiffness of the different targets. For a rigid target, $K_{\kappa }\to \infty$; hence, the second term in the brackets vanishes.
